# An interpretable ^18^F-FDG PET/CT-based radiomics model for predicting sub-3cm solitary adrenal metastases in cancer patients

**DOI:** 10.3389/fonc.2025.1691842

**Published:** 2025-11-27

**Authors:** Wenfeng Feng, Xingjian Wang, Haifeng Cai, Shunxiang Liu, Chunling Liu, Yaqi Wang, Jingwu Li, Yongliang Liu, Lixiu Cao

**Affiliations:** 1 Department of Medical Imaging, The Second Hospital of Hebei Medical University, Shijiazhuang, Hebei, China; 2 Department of Nuclear Medicine Imaging, Tangshan People’s Hospital, Tangshan, Hebei, China; 3 North China University of Science and Technology, Tangshan, Hebei, China; 4 Department of Breast Surgery, Tangshan People’s Hospital, Tangshan, Hebei, China; 5 Department of Pathology, Tangshan People’s Hospital, Tangshan, Hebei, China; 6 Department of Central Laboratory, Hebei Key Laboratory of Molecular Oncology, Tangshan, Hebei, China; 7 Department of Neurosurgery, Tangshan People’s Hospital, Tangshan, Hebei, China

**Keywords:** adrenal metastases, indeterminate adrenal nodules, PET/CT, radiomics, machine learning

## Abstract

**Purpose:**

To evaluate the potential of an interpretable radiomics model based on ^18^F-FDG PET/CT for predicting adrenal metastases (AMs) in cancer patients with indeterminate adrenal nodules.

**Materials and methods:**

A total of 177 patients with extra-adrenal malignancies and indeterminate adrenal nodules (74 metastases; 103 benign lesions) were included and randomly assigned to training and testing sets in a 7:3 ratio. Radiomics features were extracted separately from the CT and PET components of PET/CT examinations. Least Absolute Shrinkage and Selection Operator (LASSO) and multivariate logistic regression (LR) were used to identify independent predictive radiomics factors. Based on these features, single-modality CT, PET, and combined PET/CT radiomics models were constructed using four machine learning algorithms: Random Forest (RF), Support Vector Machine (SVM), LR, and Decision Tree (DT). The best-performing algorithm for each modality determined through cross-validation was selected to establish the final models. Model performance was assessed using the area under the receiver operating characteristic curve (AUC) and decision curve analysis (DCA). DeLong test was used to compare the AUCs between models. Internal validation of the best-performing radiomics model was conducted by bootstrapping to assess potential optimism. Shapley Additive Explanations (SHAP) was utilized to interpret the best-performing radiomics model.

**Results:**

The optimal algorithms identified were LR for the CT model and SVM for both the PET and integrated PET/CT models. In the testing set, the AUC values were 0.811 (95% CI: 0.694–0.928) for the CT model and 0.879 (95%CI: 0.789– 0.970) for the PET model. The combined PET/CT model integrating both CT and PET radiomics features achieved an AUC of 0.915 (95%CI: 0.834–0.997), which was significantly higher than that of the CT model alone (p < 0.05). DCA confirmed superior clinical utility of the combined PET/CT model across most threshold probabilities compared to the single-modality models. Bootstrap-corrected internal validation showed an optimism-corrected AUC of 0.919 (95% CI: 0.884-0.964), with minimal observed optimism (0.003, 95% CI: -0.002-0.007). SHAP analysis showed that a texture feature derived from the gray level size zone matrix of PET images was the most significant predictor of AMs.

**Conclusions:**

The interpretable radiomics model based on combined PET/CT data provides a non-invasive tool for predicting AMs in cancer patients with indeterminate adrenal nodules. By integrating features from both modalities, this approach significantly improves diagnostic performance and holds strong potential to support personalized treatment.

## 1 Introduction

The adrenal gland is the fourth most common site of metastasis in patients with extra-adrenal primary malignancies, with lung cancer (39%) and breast cancer (35%) representing the most frequent origins (1, 2). Among cancer patients, approximately 30% -70% of incidentally discovered adrenal masses are metastases, making adrenal metastases (AMs) the most common malignant adrenal tumors in this population (3). However, not all adrenal masses are metastases; benign adrenal lesions (ABLs) are also frequently observed. Hammarstedt et al. reported that ABLs accounted for up to 74% of adrenal lesions in patients with known extra-adrenal malignancies (4). Accurate differentiation of adrenal lesions during cancer staging or follow-up is therefore essential for guiding treatment strategies and predicting prognosis.

Although CT and MRI generally exhibit high accuracy in distinguishing benign from malignant adrenal lesions, the immediate and reliable characterization of unilateral, hyperattenuating (unenhanced CT attenuation ≥10 HU) adrenal nodules with a long diameter (LD) ≤3 cm remains challenging in patients with extra-adrenal malignancies (5–10). While histopathological examination remains the gold standard for diagnosis, adrenal biopsy is invasive, technically demanding, associated with potential complications, and not always successful (11). Furthermore, the procedure can lead to delays that impede timely clinical management. Thus, a non-invasive and efficient method for identifying AMs would provide considerable clinical benefit.

^18^F-Fluorodeoxyglucose (^18^F-FDG) positron emission tomography/computed tomography (PET/CT) is widely used in the differential diagnosis of adrenal masses (12–15). Compared to conventional anatomical imaging techniques, ^18^F-FDG PET/CT offers complementary metabolic information. Standard semi-quantitative parameters derived from PET/CT, such as the maximum standardized uptake value (SUVmax) and the ratio of adrenal tumor SUVmax to liver SUVmax (SURliver), have proven valuable in distinguishing malignant adrenal lesions (16–18). For instance, in a study by Romanisio et al. involving a heterogeneous patient population across diverse clinical settings, a SURliver cutoff of 1.5 exhibited strong diagnostic performance, achieving 100% sensitivity, 87% specificity, 73% positive predictive value, and 100% negative predictive value (19). However, these conventional metrics may not fully capture the intrinsic heterogeneity of tumors. Additionally, functional adenomas, tuberculosis, and pheochromocytoma can exhibit high uptake, potentially leading to misdiagnosis as malignant lesions (20–22). Most importantly, the aforementioned studies did not specifically focus on indeterminate adrenal lesions.

Radiomics addresses this limitation by extracting high-dimensional, mineable features from digital medical images. This methodology quantifies a vast array of features encompassing lesion shape, intensity distributions, texture patterns, and volumetric characteristics (23, 24). Previous studies have demonstrated that radiomics models based on CT or MRI alone can effectively identify adrenal metastases (AMs) in patients with indeterminate adrenal lesions (25–27). However, evidence on the application of PET/CT-derived radiomics for predicting AMs in cancer patients with indeterminate adrenal lesions remains limited. Furthermore, emerging studies suggest that integrating radiomic features from both the PET and CT components can significantly improve predictive accuracy compared to models relying on either modality alone. Importantly, the reliability, efficacy, robustness, and interpretability of predictive models are crucial for the successful clinical translation of radiomics-based approaches (28).

Therefore, this study aimed to develop and identify an optimal radiomics model based on ^18^F-FDG PET/CT to noninvasively predict AMs in cancer patients with indeterminate adrenal nodules. Additionally, we sought to enhance the interpretability of the best model using the Shapley Additive Explanations (SHAP).

### 1.1 Patients

**Figure 1:**
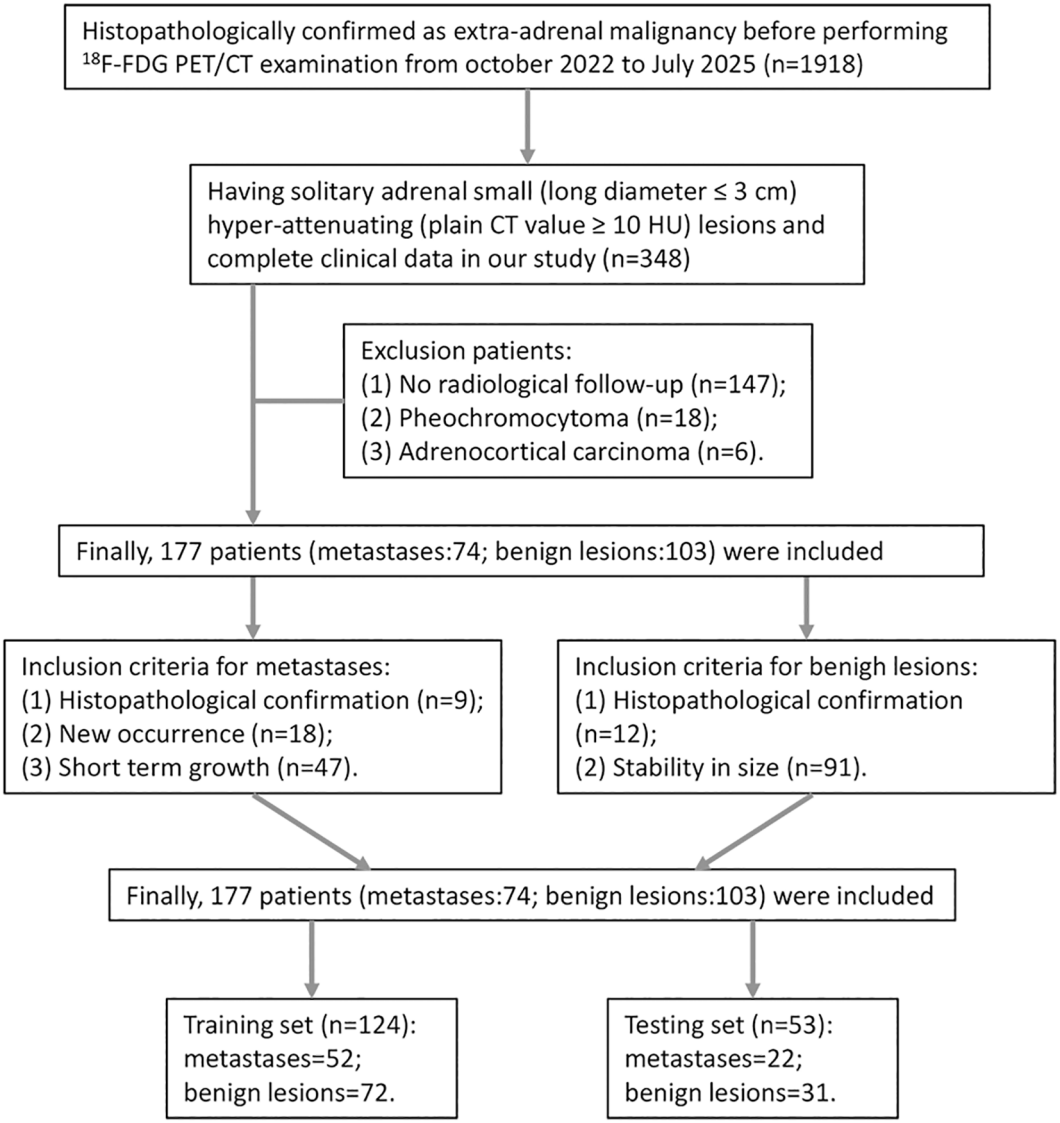
The flow diagram of sample selecting.

This retrospective study received approval from the Institutional Ethics Committee of Tangshan People’s Hospital (approval code: RMYY-LLKS-2023202). The requirement for informed consent was waived owing to the use of anonymized retrospective data. Patients who met the following criteria between October 2022 and July 2025 were included: (1) histopathologically confirmed extra-adrenal malignancy prior to ^18^F-FDG PET/CT; (2) availability of complete PET/CT imaging and clinical records; and (3) presence of indeterminate adrenal nodules defined as unilateral small (1 cm ≤ LD ≤ 3 cm) hyperattenuating (CT attenuation ≥10 HU) lesions on unenhanced CT. The reasons for the use of 1 cm as the cut-off for LD were to: (a) ensure sufficient lesion volume for reliable quantitative PET metrics, and (b) improve diagnostic confidence in distinguishing true focal lesions from imaging artifacts. Consequently, we excluded patients without radiological follow-up and those with histopathologically confirmed pheochromocytoma or adrenocortical carcinoma. Finally, AMs were confirmed in 74 patients through the following methods: histopathology (n = 9), development of new adrenal lesions (n = 18), or interval size change after treatment within a short-term period, defined as a ≥20% increase or decrease in the total sum of the adrenal lesion within a six-month period (n = 47) (29). ABLs were diagnosed in 103 patients via histopathology (n = 12) or size stability over follow-up of at least one year (n = 91). The dataset was randomly divided into training (n = 124; 72 ABLs, 52 AMs) and testing cohorts (n = 53; 31 ABLs, 22 AMs) at a ratio of 7:3 (**Figure 1**). Collected variables included age, sex, primary cancer type, as well as adrenal lesion location and size.

**Figure 2:**
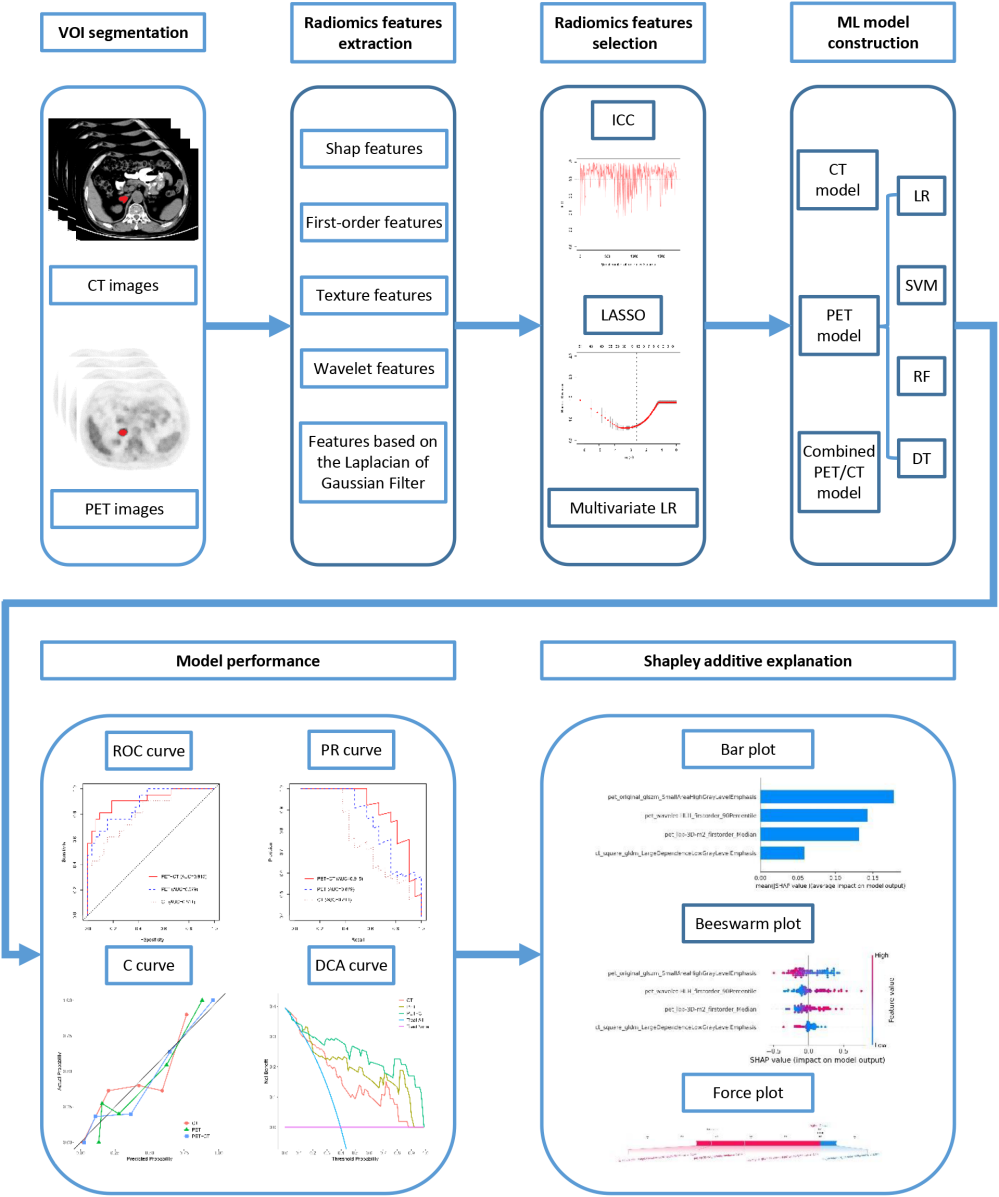
The overall workflow of this study. ICC, Intraclass correlation coefficient; LASSO, the least absolute shrinkage and selection operator; LR, logistic regression; SVM, support vector machine; RF, random forest; DT, decision tree; VOI, volumes of interest; ROC, receiver operating characteristic, PR, precision-recall; DCA, decision curve analysis.

The analytical workflow is detailed in **Figure 2**. Volumes of interest (VOIs) encompassing the adrenal tumors were manually delineated slice-by-slice on co-registered CT and PET images using 3D Slicer (version 4.11). These VOIs were subsequently exported in NIfTI format for further processing. Radiomic features were extracted from the VOIs using FAE (FeAture Explorer, version 0.6). Feature selection was performed using Least Absolute Shrinkage and Selection Operator (LASSO) regression and multivariate LR. Four machine learning classifiers were trained to distinguish AMs from ABLs, and the optimal algorithm was selected through cross-validation. Based on the optimal machine learning algorithms, three radiomic models were constructed: a CT-based model, a PET-based model, and a combined PET/CT model. The performance of each model was evaluated using the area under the receiver operating characteristic (ROC) curve (AUC), the precision-recall (PR) curve, the calibration curve, and decision curve analysis (DCA). DeLong test was applied to compare AUC values among different models, and SHAP was employed to interpret the best-performing radiomics model.

### 1.2 ^18^F-FDG PET/CT imaging protocol

Patients fasted for at least 6 hours prior to imaging, and pre-imaging blood glucose levels were confirmed to be below 11 mmol/L. All scans were performed using a Discovery Elite PET/CT system (GE Healthcare). After intravenous injection of ^18^F-FDG at a dose of 4.2 MBq/kg, patients rested quietly for approximately 60 minutes. A low-dose non-contrast CT scan was first acquired under tidal breathing from the vertex of the skull to the mid-femur, using the following parameters: 120 kVp, 80 mAs, and 5 mm slice thickness. Subsequently, PET data were collected in 3D acquisition mode from the mid-thigh to the skull vertex during shallow breathing, with an acquisition time of 2 minutes per bed position (typically 6–7 beds). Image reconstruction was performed using an ordered-subset expectation maximization (OSEM) algorithm incorporating time-of-flight and point-spread-function modelling, with attenuation correction based on the CT data.

### 1.3 Feature extraction

Two radiologists (with 3 and 5 years of PET/CT experience, respectively) independently delineated VOIs on PET and CT images using 3D Slicer (version 4.11), blinded to all clinical and histopathological information. PET-based contours were drawn along regions of elevated ^18^F-FDG avidity, while CT contours were traced along anatomical boundaries. All segmentations explicitly included necrotic, cystic, and hemorrhagic components. Discrepancies between segmentations were resolved through consensus review with a senior radiologist possessing 10 years of experience, whose decision was considered final. The resulting VOIs were exported as separate NIfTI files (PET masks and CT masks). Radiomic feature extraction was performed using FAE (FeAture Explorer, v0.6.2) under a standardized preprocessing pipeline. A total of 1,781 features were extracted from each of the PET and CT VOIs. Inter-observer reproducibility of feature extraction was evaluated using intraclass correlation coefficient (ICC) analysis in a randomly selected subset of 30 patients. Features were categorized as poor (ICC < 0.50), moderate (ICC = 0.50–0.79), or excellent (ICC ≥ 0.80) consistency. Only features with excellent agreement in the consensus VOIs were retained for subsequent modeling.

### 1.4 Radiomic machine learning model construction

The dataset was randomly split into training and testing sets in a 7:3 ratio. From the training set, radiomic features demonstrating excellent reproducibility (ICC ≥ 0.8) were first selected separately from CT and PET images. Least Absolute Shrinkage and Selection Operator (LASSO) regression was then applied for dimensionality reduction. The retained features from each imaging modality (CT-only, PET-only, and combined PET/CT) were then independently incorporated into separate multivariate logistic regression (LR) models to identify independent predictive features. We compared four machine learning algorithms—random forest (RF), support vector machine (SVM), LR, and decision tree (DT)—for their ability to predict AMs. Each algorithm was used to develop three distinct models based on PET features, CT features, and combined PET/CT features, respectively. The mean AUC derived from cross-validation was used to evaluate predictive performance and select the optimal algorithm in the training set. Based on this comparison, three final radiomic models were constructed using the best-performing algorithm: a PET-based model, a CT-based model, and a combined PET/CT-based model.

### 1.5 Bootstrap optimism correction for internal validation

Internal validation estimates a model’s predictive performance by applying it to bootstrap samples drawn from the original cohort, reflecting its expected performance in a similar population. In this study, the top-performing radiomics model was internally validated using optimism-corrected bootstrapping-a resampling technique involving random sampling with replacement from the original dataset to derive statistical inferences (30). Optimism, a form of bias, arises when a model is evaluated on the same data used for training. It is quantified as the difference between the apparent performance (measured in the bootstrap sample) and the test performance (measured in the original sample) (30). Here, the model was applied to 1000 bootstrap samples, each of size n=177, matching the original cohort. Discrimination was assessed via the AUC for each sample, and the optimism was computed accordingly. Finally, optimism and the optimism-corrected AUC for the radiomics model were calculated and reported.

### 1.6 Model interpretation

The SHAP technique offers an interpretable framework that enables clinicians to comprehend machine learning model predictions, improving transparency through both global and local explainability (31). This approach ranks features based on their importance, indicating greater contribution to model predictions for higher-ranked features. SHAP value plots further visualize the direction and magnitude of each feature’s influence. In this study, we used summary SHAP plots to identify the most impactful features within the final optimized model. Additionally, representative cases were selected to generate SHAP force plots, aiding in the detailed interpretation of the model’s decision-making process.

### 1.7 Statistical analysis

All statistical analyses and modeling were performed using R (version 4.2.1; http://www.R-project.org/) and Python (version 3.12.0; https://www.python.org/). Categorical variables were compared between AMs and ABLs using Fisher’s exact test or the chi-square test, as appropriate. Continuous variables were compared using the Mann–Whitney U test. Interobserver reproducibility of radiomic features was quantified with ICC. The following R packages were employed for technical implementation: “glmnet” for LASSO regression, “rms” for LR, “e1071” for SVM, “randomForest” for RF, “rpart” for DT, “reportROC” for ROC analysis, “pROC” for the DeLong test, “ggplot2” for DCA, and “ROCR” for PR curve analysis. SHAP analysis was conducted using the “shap” module in Python. A two-sided p-value < 0.05 was considered statistically significant.

## 2 Results

### 2.1 Clinical characteristics and performance of the clinical model

The clinical characteristics of the patients included in this study are summarized in **Table 1**. No significant differences in age, tumor size, and tumor location were observed between AMs and ABLs in either the training or testing datasets. However, significant differences were found in sex and primary cancer type between AMs and ABLs in both datasets (p < 0.05). The clinical model was developed based on sex and primary cancer type with four machine learning algorithms: LR, RF, SVM, and DT. In the testing dataset, the AUC values for these models were 0.633, 0.616, 0.629, and 0.621, respectively. Due to its marginally superior performance, LR was selected as the optimal algorithm for the clinical prediction model.

**Table 1:** Clinical characteristics of patients.

Characteristic	Training dataset		*P* value	Testing dataset		*P* value
	AMs (n=53)	ABLs (n=71)		AMs (n=21)	ABLs (n=32)	
Sex			0.045			0.034
Male	43(81.13%)	46(64.79%)		16(76.19%)	15(46.87%)	
Female	10(18.87%)	25(35.21%)		5(23.81%)	17(53.13%)	
Age (year)	66.0(60.0,70.0)	67.0(60.5,72.0)	0.360	61.0(60.0,68.0)	64.5(56.8,69.0)	0.813
Location of adrenal tumor			0.139			0.075
Right	20(37.74%)	18(25.35%)		11(52.38%)	9(28.13%)	
Left	33(62.26%)	53(74.65%)		10(47.62%)	23(71.87%)	
Size of adrenal tumor						
Long diameter(cm)	1.50(1.20,2.20)	1.40(1.20,1.80)	0.359	1.60(1.40,2.40)	1.35(1.20,1.70)	0.077
Short diameter(cm)	1.20(1.00,1.60)	1.20(1.00,1.60)	0.824	1.20(1.00,1.30)	1.15(1.00,1.42)	0.703
Types of primary cancer			0.031			0.048
Lung cancer	32(60.38%)	29(40.85%)		15(71.43%)	14(43.75%)	
Non-lung cancer	21(39.62%)	42(59.15%)		6(28.57%)	18(56.25%)	

AMs, adrenal metastases; ABLs, adrenal benign lesions.

### 2.2 Extracting and screening radiomic features

**Figure 3:**
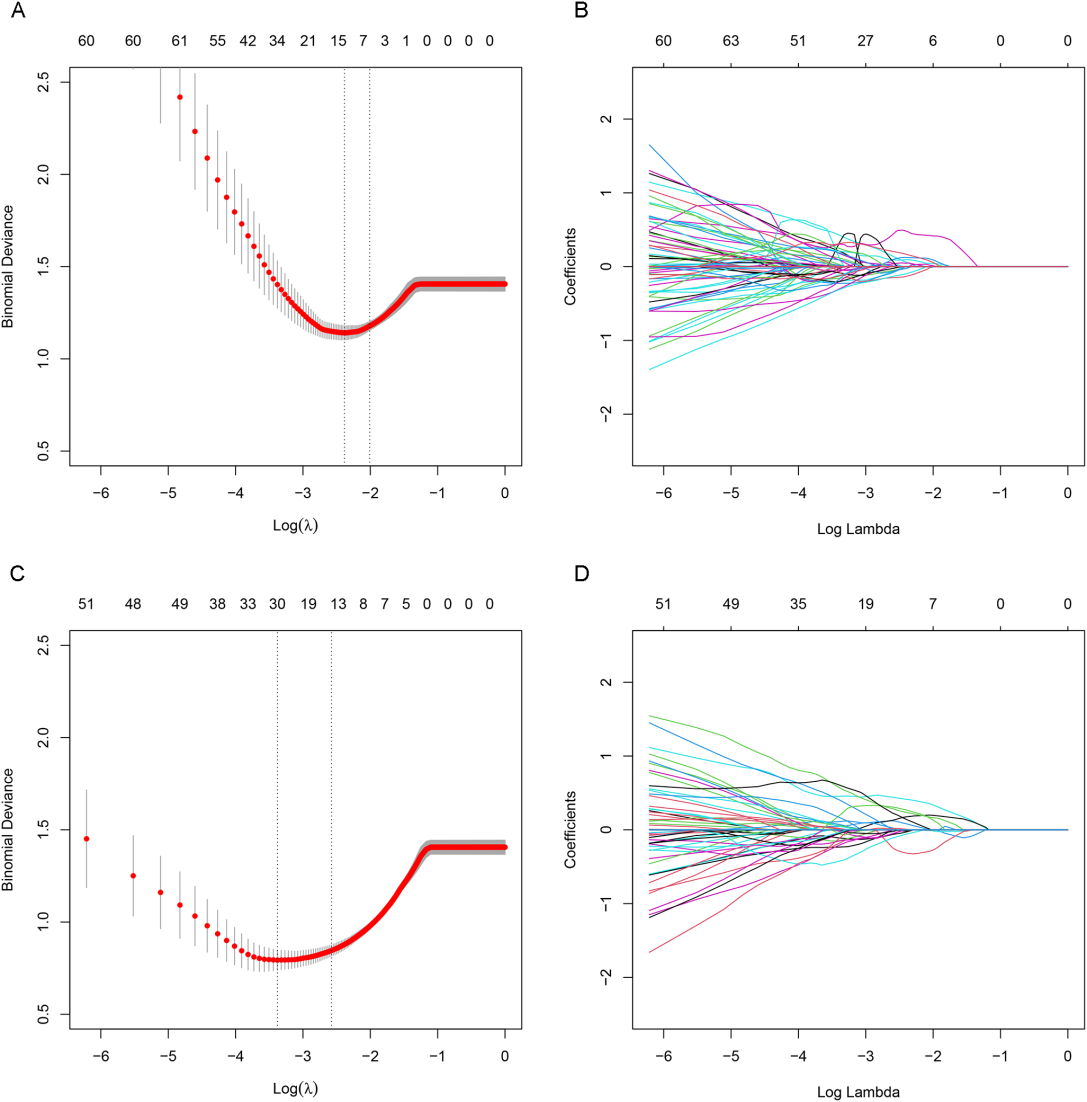
LASSO regression selected 9 CT features **(A, B)**; LASSO regression selected 15 PET features **(C, D)**.

A total of 1,781 radiomic features were extracted from CT and PET images, respectively. Following inter-observer reproducibility assessment, 1,451 CT features and 1,606 PET features with excellent reproducibility (ICC ≥ 0.8) were retained for further analysis. The mean ICC values were 0.901 for the remaining CT features and 0.921 for PET features (**Supplementary Figures S1A, B**). 9 CT and 15 PET features were selected using LASSO regression, respectively (**Figures 3A–D).** And then multivariate LR identified 3 CT-based, 4 PET-based, and 4 combined PET/CT-based radiomic predictors. The correlation coefficients among the radiomic predictors within each feature set (CT, PET, and PET/CT) were relatively low (**Supplementary Figures S2A–C**), indicating minimal multicollinearity.

### 2.3 Performance of machine learning algorithms and radiomic models

**Figure 4:**
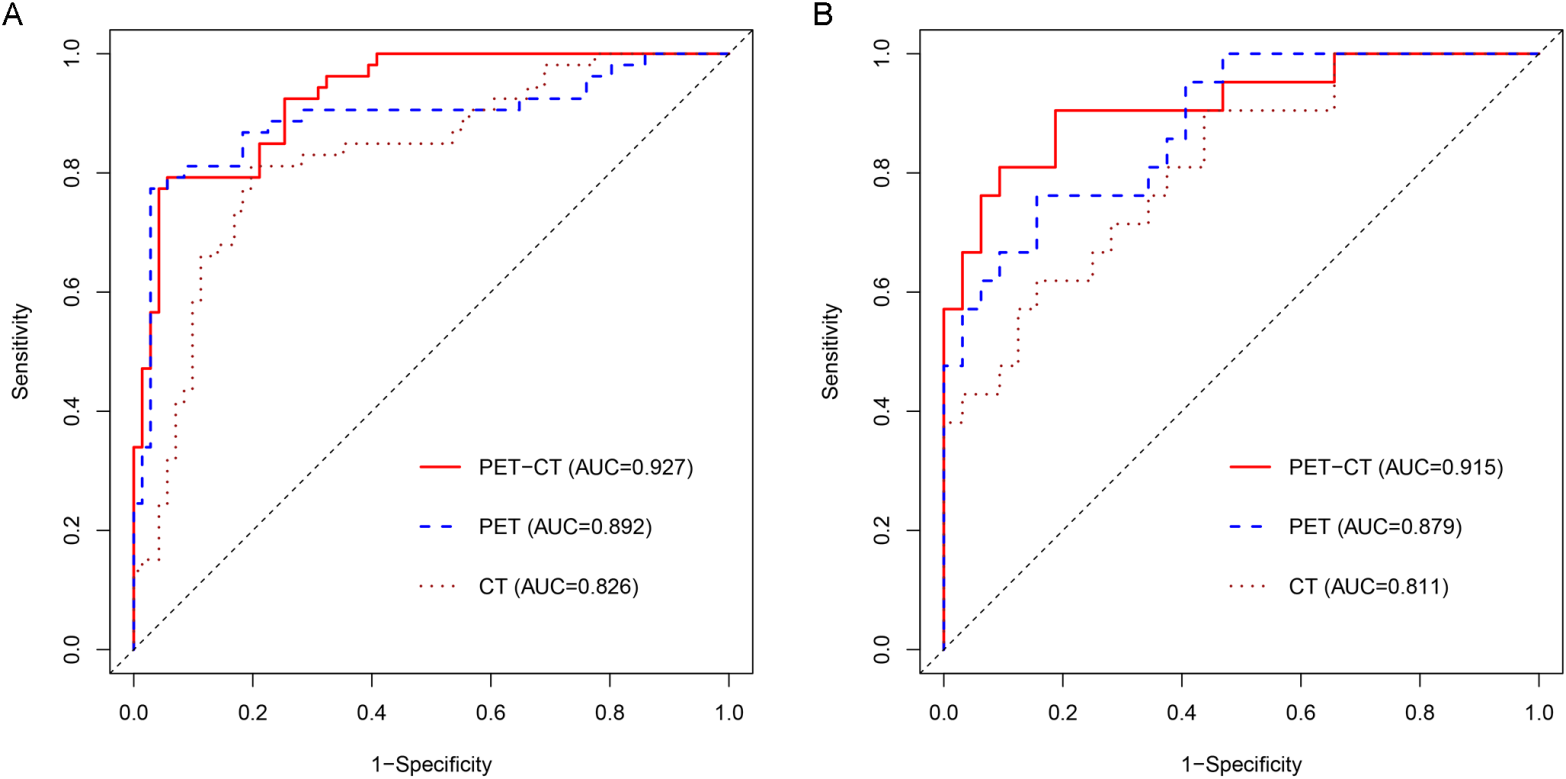
The ROCs of the three final radiomics models developed using the optimal machine learning algorithm in the training **(A)** and testing **(B)** datasets.

**Figure 5:**
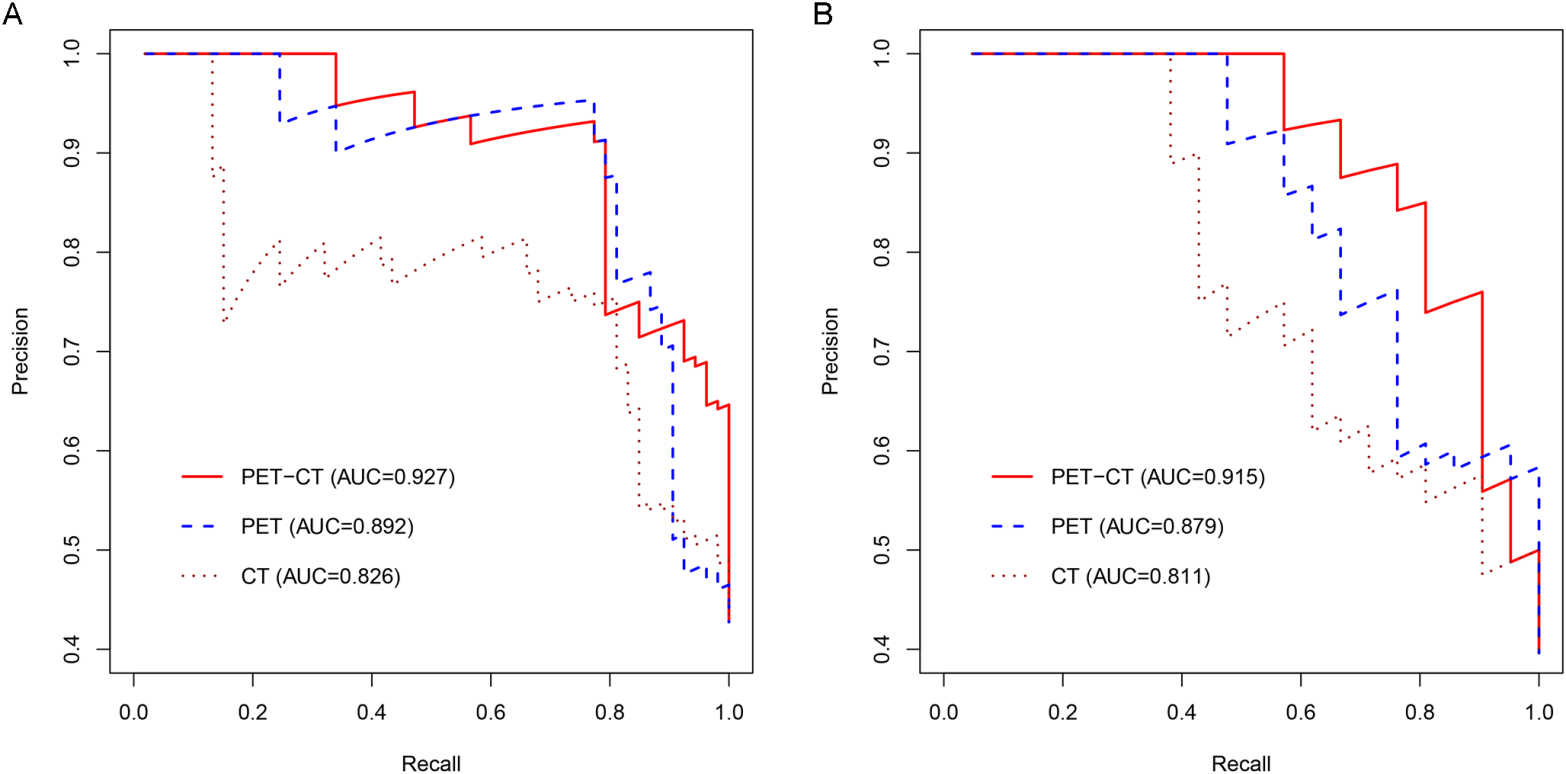
The PR curves of the three final radiomics models developed using the optimal machine learning algorithm in the training **(A)** and testing **(B)** datasets.

**Figure 6:**
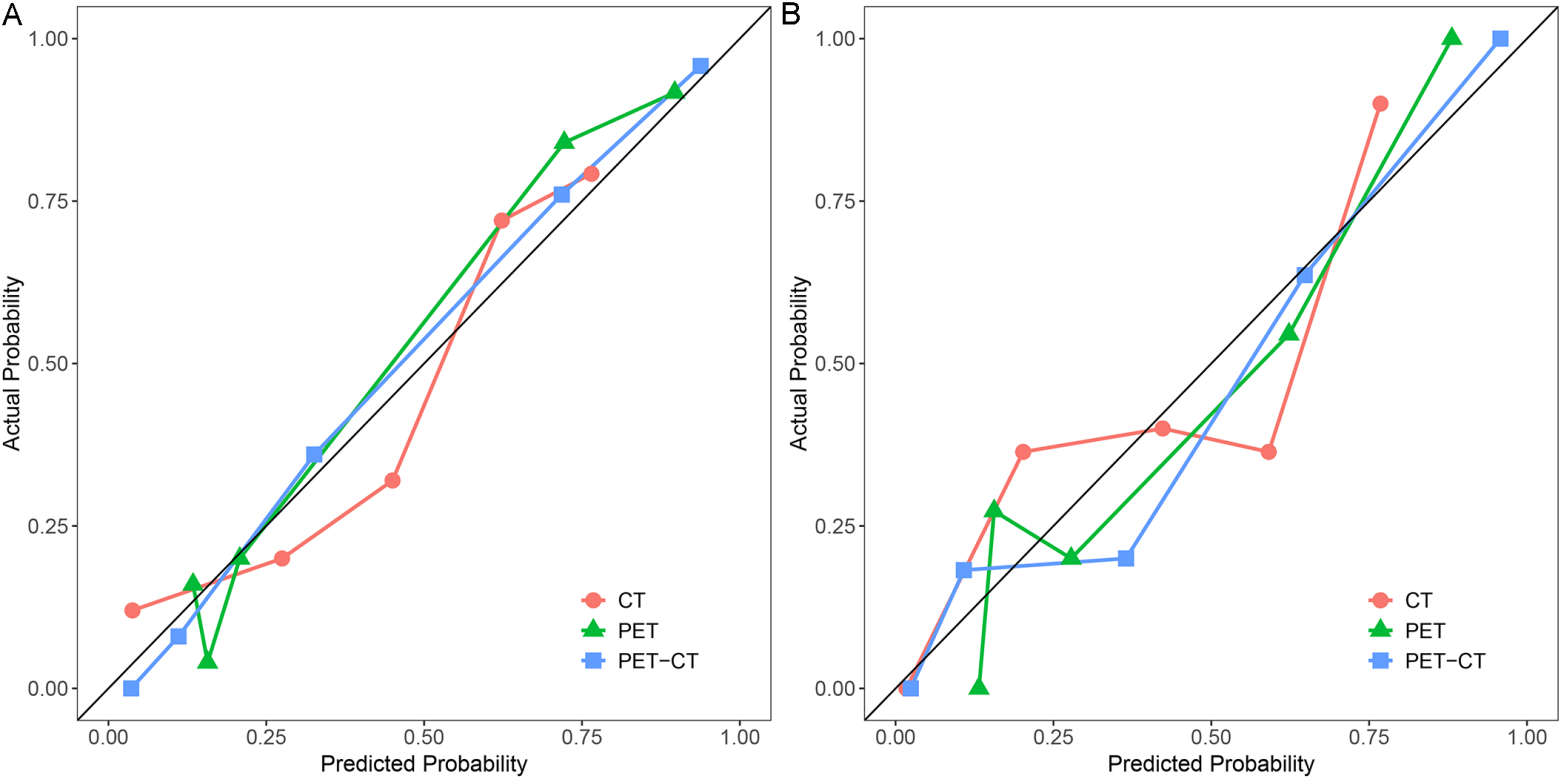
The three final radiomics models had good calibration curves in both the training **(A)** and testing **(B)** datasets.

**Figure 7:**
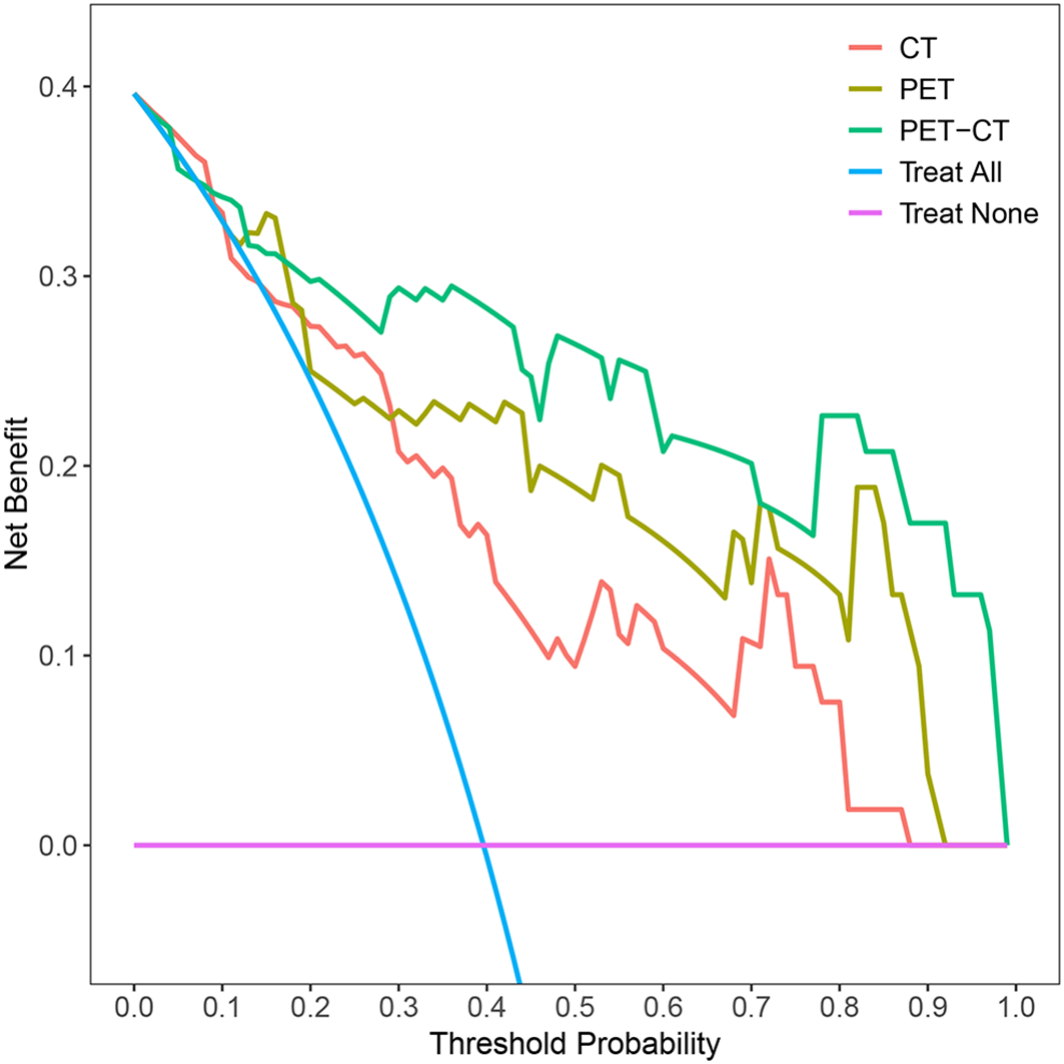
DCA showed that the combined PET/CT radiomics model provided the highest net benefit across threshold probabilities ranging from approximately 0.2 to 1.0 in the testing dataset.

To predict AMs, we constructed CT-, PET-, and combined PET/CT-based radiomic models using four machine learning algorithms: LR, SVM, RF, and DT. Based on 5-fold cross-validation within the training set, LR achieved the highest mean AUC (0.766) for the CT model, whereas SVM performed best for both the PET model (mean AUC = 0.849) and the combined PET/CT model (mean AUC = 0.874) (**Table 2**). The best-performing algorithm for each modality was subsequently used to build three final predictive models. The combined PET/CT model showed the highest predictive accuracy, with AUCs of 0.927 (95% CI: 0.885–0.970) in the training set and 0.915 (95% CI: 0.834–0.997) in the testing set. The PET model achieved AUCs of 0.892 (95% CI: 0.825–0.958) in training and 0.879 (95% CI: 0.789–0.970) in testing sets, while the CT model yielded AUCs of 0.826 (95% CI: 0.750–0.901) and 0.811 (95% CI: 0.694–0.928), respectively (**Table 3**, **Figure 4**). PR curves further confirmed the robust diagnostic accuracy and reliability of the combined PET/CT model in both datasets, with curves approaching the upper-right corner (**Figure 5**). Calibration curves indicated good agreement between predicted probabilities and actual outcomes across all models in training and testing sets (**Figure 6**). DCA demonstrated that the combined PET/CT model offered a higher net benefit across most threshold probabilities compared to other models, with the greatest net benefit within the threshold probability range of 0.2–1.0 in the testing set (**Figure 7**). The DeLong test confirmed that the combined PET/CT model significantly outperformed both the clinical model (p < 0.001) and the CT model (p = 0.045) (**Table 4**).

**Table 2:** Comparison of the predictive performance of four machine learning algorithms for AMs using CT, PET, and combined PET/CT radiomic features based on cross-validated mean AUC values.

ML algorithm	Cross-validation, mean AUC		
	CT	PET	Combined PET/CT
LR	0.766	0.848	0.872
SVM	0.760	0.849	0.874
RF	0.617	0.786	0.800
DT	0.607	0.776	0.780

ML, machine learning; AUC, The area under the curve; LR, Logistic Regression; SVM, Support Vector Machine; RF, Random Forest; DT, Decision tree.

**Table 3:** Predictive performance of the three final radiomics models for AMs using the optimal machine learning algorithm.

Model	Dataset	Accuracy	Sensitivity	Specificity	F1score	AUC(95%CI)
CT	Training	0.806	0.811	0.803	0.782	0.826(0.750,0.901)
	Testing	0.698	0.905	0.562	0.704	0.811(0.694,0.928)
PET	Training	0.887	0.774	0.972	0.854	0.892(0.825,0.958)
	Testing	0.811	0.862	0.844	0.762	0.879(0.789,0.970)
Combined PET/CT	Training	0.879	0.792	0.944	0.849	0.927(0.885,0.970)
	Testing	0.849	0.905	0.812	0.826	0.915(0.834,0.997)

AUC, the area under the curve; CI, confidential interval.

**Table 4:** Comparison of AUCs between different models in the testing dataset.

Models	AUC	Z statistic	P
Combined PET/CT model *vs* PET model	0.915 *vs* 0.879	1.05	0.293
Combined PET/CT model *vs* CT model	0.915 *vs* 0.811	2.00	0.045
Combined PET/CT model *vs* Clinical model	0.915 *vs* 0.633	3.41	<0.001

AUC, the area under the curve.

### 2.4 Internal validation of the combined PET/CT model

The combined PET/CT model underwent internal validation using 1000 bootstrap samples, each matching the original cohort size (n=177). The pooled mean optimism for the AUC was 0.003 (95% CI: -0.002-0.007), resulting in an optimism-corrected AUC of 0.919 (95% CI: 0.884-0.964). The narrow confidence interval and minimal optimism indicate stable discriminative performance of the model across samples.

### 2.5 Model interpretability exploration

**Figure 8:**
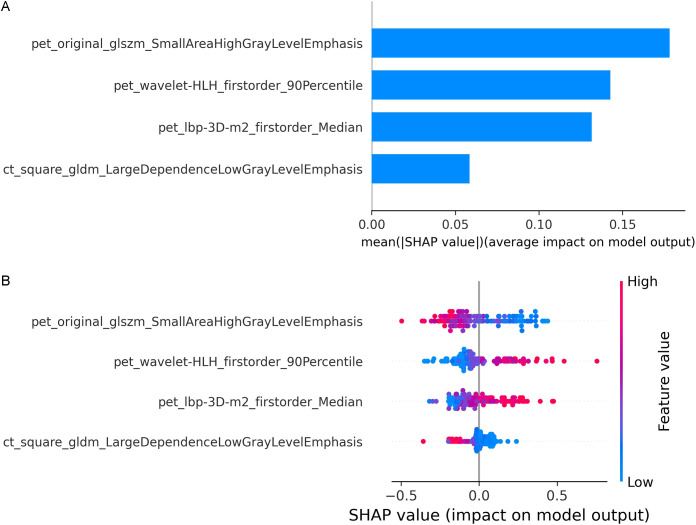
The interpretability of the best combined PET/CT radiomics model was assessed using the SHAP method. **(A)** The SHAP bar plot showed the importance of each feature based on the mean absolute SHAP values. **(B)** The SHAP beeswarm plot showed the impact of each feature on the model predictions.

**Figure 9:**
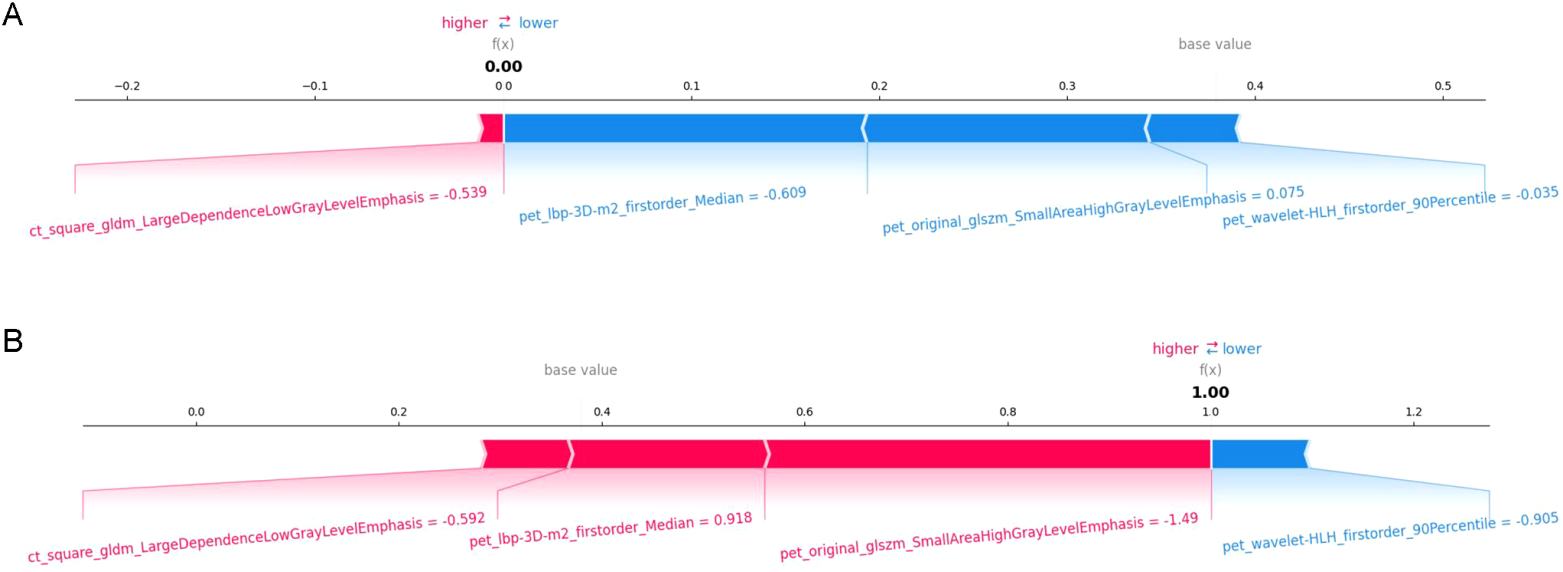
Two representative cases correctly predicted as ABL **(A)** and AM **(B)** were individually visualized using the SHAP force plots. These plots illustrated prediction related features of individual patients and contributions to the predicted probability of AMs, with SHAP values quantifying the magnitude and direction of each feature’s effect. The bolded number represented the model’s raw prediction output (f(x)). Features in red (on the left) indicated those increasing the predicted probability of AMs, while features in blue correspond to a decrease in probability. The length of each arrow reflected the magnitude of the effect on the prediction. The longer the arrow the larger the effect.

SHAP values were computed for each radiomic feature in the combined PET/CT model. In the global interpretation, the SHAP bar plot (**Figure 8A**) illustrated the importance of each feature to the model predictions, where a larger absolute SHAP value indicated a stronger influence on the model output. For instance, pet_original_glszm_SmallAreaHighGrayLevelEmphasis was the most influential feature, followed by pet_wavelet-HLH_firstorder_90Percentile, pet_lbp-3D-m2_firstorder_Median, and ct_square_gldm_LargeDependenceLowGrayLevelEmphasis. The SHAP beeswarm plot (**Figure 8B**) visualized the relationship between feature values and their corresponding SHAP values. In the plot, red dots represent higher feature values, and blue dots indicate lower values. The x-axis corresponds to the magnitude of the SHAP value. Notably, lower values of pet_original_glszm_SmallAreaHighGrayLevelEmphasis and ct_square_gldm_LargeDependenceLowGrayLevelEmphasis were associated with an increased predicted probability of AMs. Conversely, lower values of pet_wavelet-HLH_firstorder_90Percentile and pet_lbp-3D-m2_firstorder_Median were associated with a decreased predicted probability of AMs. **Figures 9A, B** present the force plots illustrating personalized feature attributions for two representative patients from the study cohort. The red arrows indicate features that push the prediction toward metastasis (positive contribution), whereas blue arrows represent features that push the prediction toward benign (negative contribution). The length of each arrow is proportional to the SHAP value of the feature. As shown in **Figure 9A**, the output value for this patient was 0.00, which was lower than the base value, suggesting a prediction of benign. Among these features, pet_lbp-3D-m2_firstorder_Median (value: -0.609) had the greatest influence in driving the prediction toward benign, followed by pet_original_glszm_SmallAreaHighGrayLevelEmphasis (value: 0.075) and pet_wavelet-HLH_firstorder_90Percentile (value: -0.035). In contrast, ct_square_gldm_LargeDependenceLowGrayLevelEmphasis (value: -0.539) contributed only marginally toward the prediction of metastasis. In **Figure 9B**, the output value for the other patient was 1.00, which was higher than the base value, supporting a prediction of metastasis. Here, pet_original_glszm_SmallAreaHighGrayLevelEmphasis (value: -1.49) contributed most strongly to the prediction of metastasis, followed by pet_lbp-3D-m2_firstorder_Median (value: 0.918) and ct_square_gldm_LargeDependenceLowGrayLevelEmphasis (value: -0.592). Meanwhile, pet_wavelet-HLH_firstorder_90Percentile (value: -0.905) had only a minor effect in favoring a benign prediction.

## 3 Discussion

In patients with extra-adrenal malignancies, both AMs and ABLs are common tumors. When an adrenal lesion is identified during cancer staging or follow-up, accurate diagnosis is extremely critical for determining optimal treatment strategies. PET/CT, which effectively integrates metabolic (PET) and anatomical (CT) imaging, is widely used in this clinical context. Radiomics, an emerging advanced image analysis technique, enables high-throughput extraction of quantitative features from medical images. Its non-invasive and repeatable nature offers great potential for characterizing tumor heterogeneity. In this study, the combined PET/CT radiomics model developed with the SVM algorithm demonstrated superior performance in discriminating AMs from ALBs in cancer patients with indeterminate adrenal nodules, outperforming both single-modality imaging models and clinical model.

In cancer patients with indeterminate adrenal nodules, previous studies have indicated that conventional imaging features and metabolic parameters possessed some value in the differential diagnosis of AMs. For example, Cao et al. reported that a non-contrast CT attenuation value > 27.5 HU was identified as an independent predictor of AMs in lung cancer patients, with an AUC of 0.789 (29). Similarly, our previous PET/CT study identified a non-contrast CT attenuation threshold > 32.5 HU and a SURliver > 1.493 as independent predictors of AMs in patients with extra-adrenal malignancies, with AUCs of 0.788 and 0.851, respectively (32). Furthermore, Romanisio et al. demonstrated that a SURliver cutoff of 1.5 performed well in predicting adrenal malignancy within a heterogeneous cohort spanning multiple clinical settings (endocrinological, oncological, and surgical), achieving an AUC of 0.868 (19). However, the conventional imaging features and metabolic parameters capture only a limited portion of the tumor’s information. The often modest differences in these parameters between AMs and ABLs lead to suboptimal diagnostic performance. The emergence of radiomics has significantly improved the utilization of information in images. In a systematic review, Crimì et al. highlighted that, despite methodological heterogeneity, CT texture analysis represents a promising tool for the initial evaluation of adrenal lesions (33). For example, Torresan et al. found that radiomics with CT texture analysis achieved high diagnostic accuracy-with both sensitivity and specificity exceeding 90%-in discriminating malignant from benign adrenocortical tumors (34). Moreover, recent studies have demonstrated that radiomics models based on non-contrast CT images achieved AUCs ranging from 0.888 to 0.935 in the testing datasets for predicting AMs within this patient population, outperforming conventional non-contrast CT attenuation values (25, 26, 35). This study is consistent with previous findings. Our CT-based and PET-based radiomics models based on PET/CT images achieved AUCs of 0.811 and 0.879, respectively, in the testing dataset for predicting AMs in cancer patients with indeterminate adrenal nodules. These results surpassed those obtained using conventional imaging features, further supporting the value of radiomics in extracting more diagnostic information from medical images. However, the performance of our CT radiomics model was somewhat lower than that reported in previous radiomics studies based on non-contrast CT. This discrepancy may be due to the use of thicker-slice CT images and relatively limited sample size in our study, both of which may affect the performance of the models (36).

Furthermore, previous studies have indicated that multi-modal integration can substantially improve predictive performance (37–39). For example, Yin et al. used stacked generalization to combine PET and CT imaging information, improving the AUC for predicting EGFR mutation status in lung adenocarcinoma patients from 0.72 and 0.74 (single-modality models) to 0.84 (combined PET/CT model) (36). Given the technical challenges in PET/CT image registration for small indeterminate adrenal nodules (LD ≤ 3 cm), we independently extracted radiomic features from PET and CT images and subsequently integrated these feature sets. Our results showed that integrating PET and CT information significantly improved the AUC in the testing set from 0.811 (CT model) and 0.879 (PET model) to 0.915. These findings confirmed that multi-modal feature fusion can further enhance the prediction of AMs in cancer patients. To evaluate the robustness of the combined PET/CT model, we performed internal validation using the standard bootstrap method. The model retained strong discriminative performance, with an optimism-corrected AUC of 0.919 (95% CI: 0.884-0.964). Nevertheless, external validation in an independent cohort is warranted before clinical implementation can be considered.

SVM, LR, RF, and DT are the most widely used machine learning algorithms in radiomics research (26, 28, 40). Since no single algorithm is universally optimal for all medical problems (41, 42), comparing different algorithms is essential to identify the most appropriate model for a specific clinical problem. In this study, we constructed radiomics models using these four algorithms (SVM, LR, RF, DT) based on CT, PET, and combined PET/CT feature sets. The results demonstrated that the LR-based radiomics model achieved the best performance in CT features (mean AUC = 0.766), whereas the SVM-based models performed best for both PET features (mean AUC = 0.849) and the combined PET/CT feature set (mean AUC = 0.874). These findings indicated that LR was the optimal algorithm for characterizing indeterminate adrenal nodules based on CT images alone, while SVM was optimal for models utilizing PET features or the combined PET/CT feature set in this study. Therefore, we boldly speculate that SVM or LR may be the preferred algorithm in the future radiomics research of AMS algorithm of choice in future radiomics studies of AMs, and its choice is determined by the imaging mode analyzed (CT, PET, or combined PET/CT).

The inherent “black-box” nature and limited interpretability of machine learning models hinder their widespread application in clinical practice, as clinicians are often reluctant to rely on predictions without clear rationale. The introduction of SHAP has improved interpretability in radiomics and machine learning by providing transparent insights into model decisions (43, 44). In this study, SHAP analysis revealed that the PET-derived feature “original_glszm_SmallAreaHighGrayLevelEmphasis” had the most significant influence on model prediction. Higher values of this feature were associated with a decreased predicted probability of AMs, shifting predictions toward ABLs. Conversely, adrenal nodules with lower values of this feature were more frequently classified as AMs. Gray-Level Size Zone Matrix (GLSZM) features are commonly used to characterize image texture and tumor heterogeneity by quantifying the spatial relationships between groups of pixels or voxels with similar intensity values. In a related context, a recent study reported that lower values of the CT-derived feature “wavelet_HLL_glszm_SmallAreaLowGrayLevelEmphasis” were predictive of adrenocortical carcinomas (45), which was generally consistent with our findings. The GLSZM SmallAreaHighGrayLevelEmphasis reflects heterogeneity of glucose uptake. In malignant adrenal lesions (such as metastases and adrenocortical carcinomas), this heterogeneity tends to be more pronounced, likely due to a more aggressive tumor microenvironment. To further enhance local interpretability, we visualized individual predictions using SHAP force plots, illustrating how specific features contribute to each case’s prediction. This method helps clarify the internal decision-making process of machine learning models, promoting clinical trust and facilitating their integration into diagnostic workflows.

Our study has several limitations that should be acknowledged. First, it was a single-center study with a limited sample size and a comparatively high proportion of ABLs. The restricted sample size may account for the lack of significant differences observed between the performance of the combined PET/CT model and the PET model in the testing dataset. A future multi-center study with a larger cohort would help enhance the generalizability of the models. Second, the diagnosis of some adrenal nodules in this study relied solely on imaging follow-up, which reflects current clinical practice but may introduce diagnostic uncertainty. Third, the CT and PET images analyzed were thick-slice, and tumor vascularity was not evaluated. The use of thin-slice contrast-enhanced CT in future studies may improve model performance. Fourth, our analysis utilized only 3D VOI. Although 3D VOI have been reported to capture tumor heterogeneity more comprehensively than 2D regions of interest (ROI) (46), 2D ROI offer greater practicality and ease of use, which may facilitate clinical adoption. Fifth, the potential influence of the hormonal secretion pattern of adrenal adenomas was not evaluated in this study. Existing literature reports discordant findings regarding glucose uptake in functioning versus non-functioning adenomas (47, 48). Investigating the interplay between endocrine status and radiomic features represents an important goal for future studies. Finally, the radiomics models developed in this study lacked external validation. Although internal validation using bootstrapping was performed to assess potential optimism of the optimal model and its optimism-corrected AUC, multi-center collaboration is necessary to further validate and improve the predictive accuracy and generalization ability of these models.

In conclusion, the SVM-based model integrating radiomic features from both CT and PET images provides a non-invasive and effective approach for predicting AMs in cancer patients with indeterminate adrenal nodules.

## Data Availability

The raw data supporting the conclusions of this article will be made available by the authors, without undue reservation.
