# Supplementary material for: An interpretable 18F-FDG PET/CT-based radiomics model for predicting sub-3cm solitary adrenal metastases in cancer patients
Source: Front Oncol. 2025 Nov 27;15:1691842. doi: 10.3389/fonc.2025.1691842 (PMC12695529; doi:10.3389/fonc.2025.1691842)
Supplement: Supplementary file 1 [file DataSheet1.docx]

Supplementary Material

# Supplementary Figures


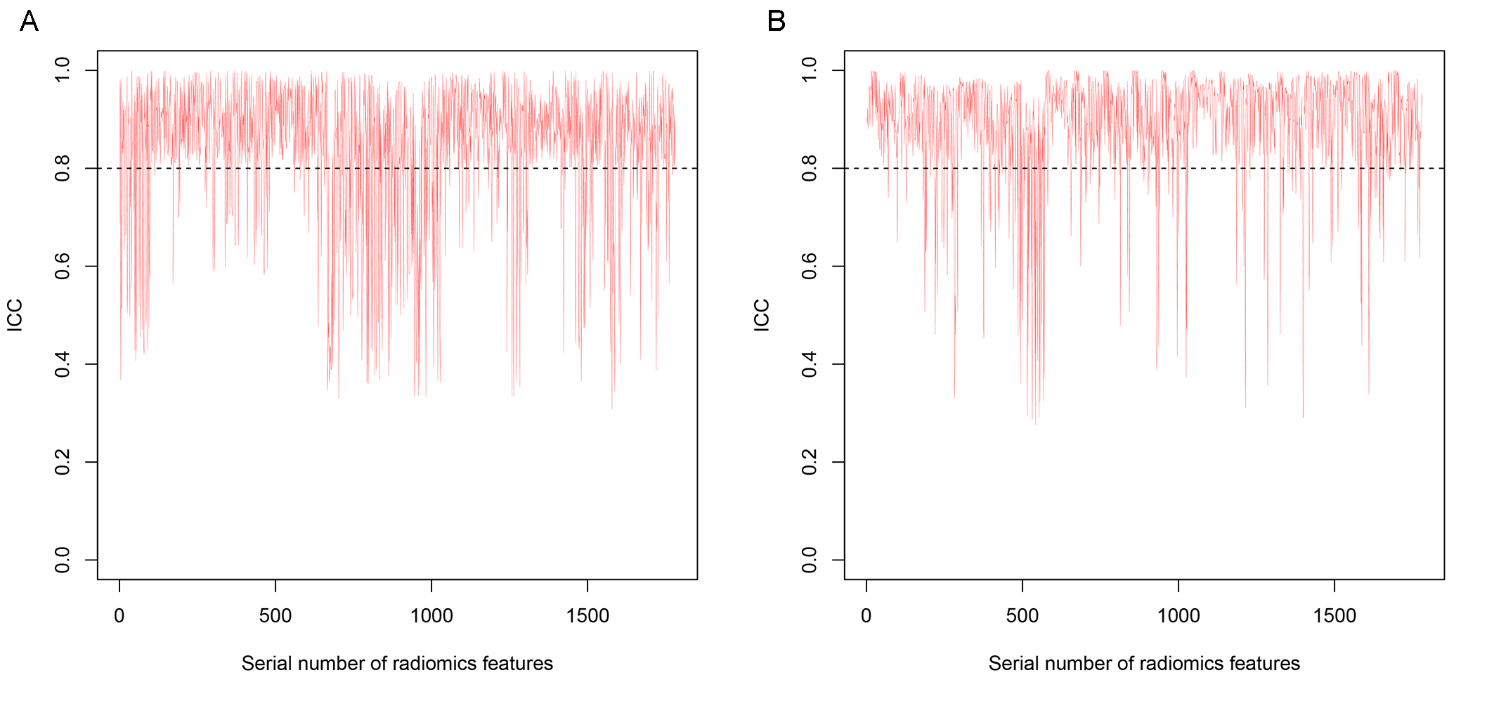
**Supplementary Figure 1.** (A) The mean ICC value of the remaining CT features was 0.901; (B) The mean ICC value of the remaining PET features was 0.921.


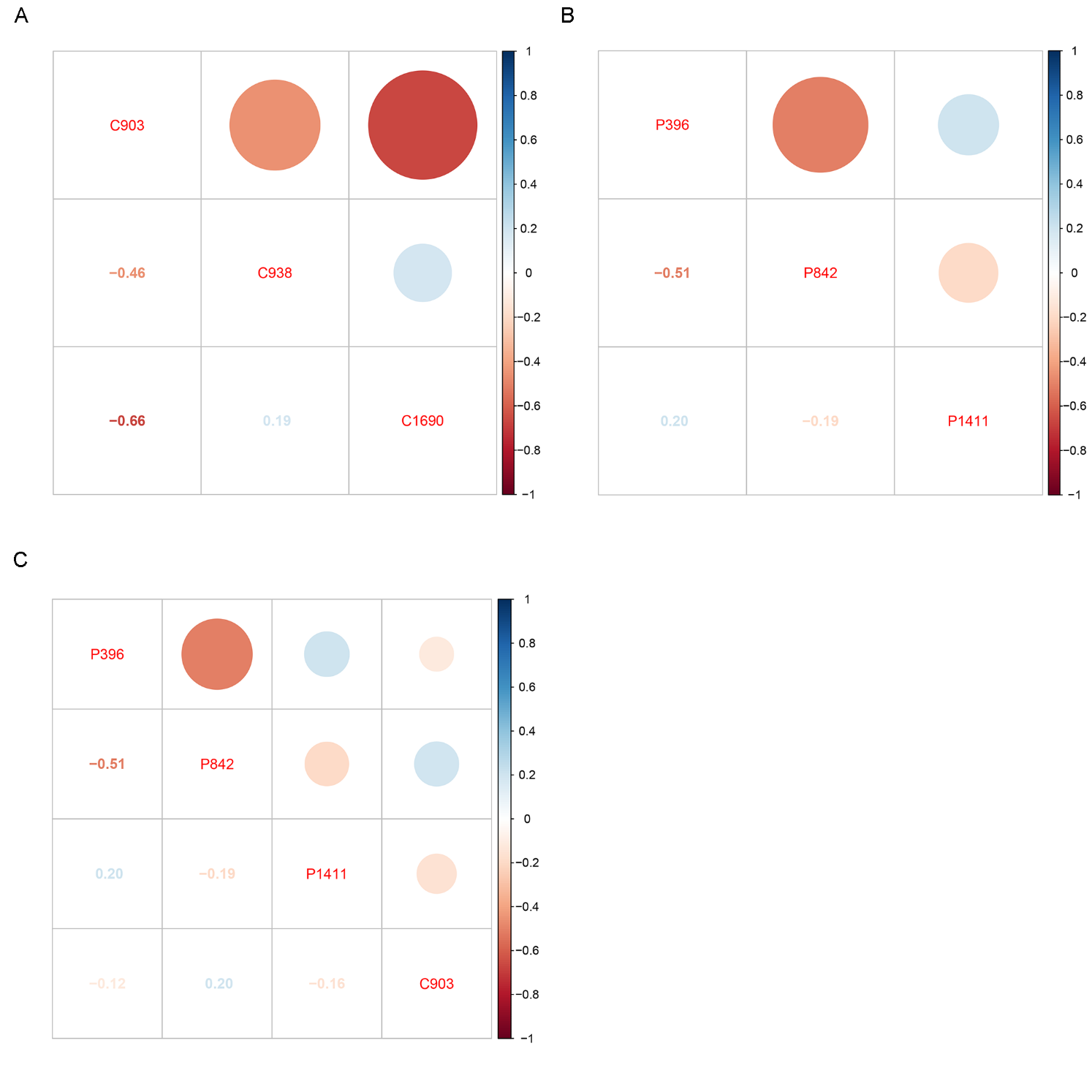
**Supplementary Figure 2.** The correlation coefficients between the independent radiomic predictors of CT model (A), PET model (B), and combined PET/CT model (C) were all relatively low.
